# Opioid modulation of GABA release in the rat inferior colliculus

**DOI:** 10.1186/1471-2202-5-31

**Published:** 2004-09-07

**Authors:** Walaiporn Tongjaroenbungam, Nopporn Jongkamonwiwat, Joanna Cunningham, Pansiri Phansuwan-Pujito, Hilary C Dodson, Andrew Forge, Piyarat Govitrapong, Stefano O Casalotti

**Affiliations:** 1 Neuro-Behavioural Biology Centre, Mahidol University Salaya Nakorn Pathom 73170 Thailand; 2 Department of Vision and Ophthalmology, King's College London, St. Thomas' Hospital, London, UK; 3 Department of Anatomy, Faculty of MedicineSrinakarinwirot University Bangkok 10110 Thailand; 4 UCL Centre for Auditory Research, University College London, 330 Grays Inn Road London WCIX 8EE UK; 5 Center for Neuroscience and Department of Pharmacology, Faculty of Science, Bangkok, Thailand

## Abstract

**Background:**

The inferior colliculus, which receives almost all ascending and descending auditory signals, plays a crucial role in the processing of auditory information. While the majority of the recorded activities in the inferior colliculus are attributed to GABAergic and glutamatergic signalling, other neurotransmitter systems are expressed in this brain area including opiate peptides and their receptors which may play a modulatory role in neuronal communication.

**Results:**

Using a perfusion protocol we demonstrate that morphine can inhibit KCl-induced release of [^3^H]GABA from rat inferior colliculus slices. DAMGO ([D-Ala(2), N-Me-Phe(4), Gly(5)-ol]-enkephalin) but not DADLE ([D-Ala2, D-Leu5]-enkephalin or U69593 has the same effect as morphine indicating that μ rather than δ or κ opioid receptors mediate this action. [^3^H]GABA release was diminished by 16%, and this was not altered by the protein kinase C inhibitor bisindolylmaleimide I. Immunostaining of inferior colliculus cryosections shows extensive staining for glutamic acid decarboxylase, more limited staining for μ opiate receptors and relatively few neurons co-stained for both proteins.

**Conclusion:**

The results suggest that μ-opioid receptor ligands can modify neurotransmitter release in a sub population of GABAergic neurons of the inferior colliculus. This could have important physiological implications in the processing of hearing information and/or other functions attributed to the inferior colliculus such as audiogenic seizures and aversive behaviour.

## Background

Sounds are first converted into neuronal signals in the inner ear and then conveyed to the cerebral cortex via a number of discrete brain areas including the inferior colliculus. Each of these areas receives ascending pathways carrying signals from one or both ears and descending pathways from higher brain centres. The current knowledge of the neurochemical events occurring at each of these brain centres is limited [1,2]. In the inferior colliculus studies have been carried out to characterise the role of GABAergic neurons especially in sound localisation which is believed to be one of the main functions of this brain area [3,4]. Additionally the inferior colliculus has ben implicated in audiogenic seizures and aversive behaviour in which GABAergic neurons may also play an important role. [5,6]

The neuronal communication occurring in the inferior colliculus is likely to be influenced by modulatory systems such as those of peptidergic neurotransmitters. Opiate receptor gene expression, immunoreactivity and activity in the inferior colliculus have been described [7-10] although detailed studies on the effect of opiate on GABA neurotransmitter release in this brain regions have not been carried out.

Three classes of opiate peptides endorphins, dynorphins and enkephalins activate μ, κ and δ-opiate receptors subtypes respectively [12]. Recently a fourth related receptor ORL1 activated by the peptide nociceptin has been identified and its distinct pharmacology has been described [13]. All opiate receptors are associated with either Go or Gi subunits and they mediate inhibitory actions including pre-synaptic inhibition of neurotransmitter release. Different mechanisms of inhibition of neurotransmitter release have been reported in various tissues and neurons [14]. For example, in the periaqueductal gray stimulation of opiate receptors and their associated G-proteins results in the activation of potassium channels [15] while in the hippocampus, inhibition of the GABAergic activity by opioid is independent of potassium channel activation [16].

In previous work we have established the presence and distribution of opiate receptors in the adult and developing rat cochlea suggesting that the opiate system has a role in hearing function [17,18]. In order to extend our knowledge of the role of opiate system in hearing it is necessary to characterise its presence and role also in the auditory pathways. Our hypothesis was that opiate peptides can modulate synaptic function in the auditory pathways by pre-synaptically altering the release of other neurotransmitters. To test this hypothesis we have used opiate drugs to inhibit the release of [^3^H]GABA from inferior colliculus slices

## Results and Discussion

### KCl-induced [^3^H]GABA release

**Figure 1:**
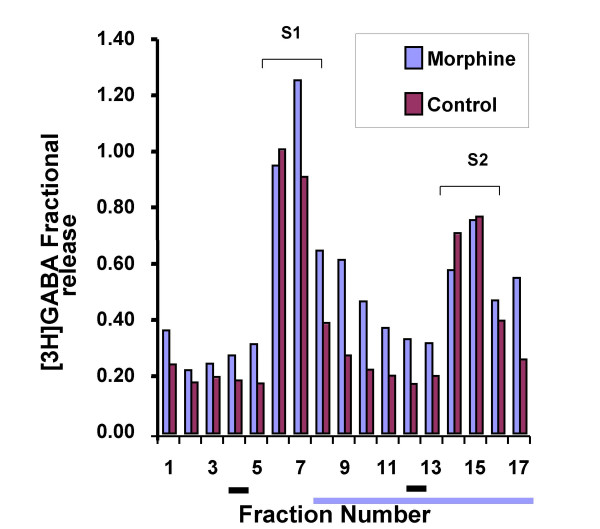
Elution profile of KCl-stimulated [^3^H]GABA release from inferior colliculus slices. Inferior colliculus slices were incubated with 11 nM [^3^H]GABA for 30 min and perfused (0.5 ml/min) with Krebs buffer for 30 min in a Brandel superfiltration apparatus. Fractions (1 ml) were then collected every 2 min. Krebs buffer containing 25 mM KCl was loaded into the system at times corresponding to fraction 4 and 12 (short black lines) which elicited [^3^H]GABA release peaks in fractions 6–7 and fraction 14–15. Krebs buffer containing modulating drugs (1 μM morphine-long blue line) was added from the time corresponding to fraction 8. Scintillation fluid (3 ml) was added to the each fractionated eluate and to the solubilized tissue samples and counted. Data are expressed as fractional release which is calculated as the radioactivity released in one fraction divided by the amount of radioactivity present in the tissue just before that fraction. The two peaks from each elution profile are referred to as S1 and S2. The data from a representative experiment are shown.

Inferior colliculus slices pre-incubated with [^3^H]GABA were perfused for 30 min and stimulated twice with 25 mM KCl to elicit neurotransmitter release. The eluate was collected in 1 ml fractions and the released radioactivity was assessed by scintillation counting. Figure 1 shows two examples of typical release profiles from slices perfused with either Krebs buffer throughout (control) or with Krebs buffer for fractions 1–7 and with Krebs containing 1 μM morphine for the remaining fractions, where both samples were stimulated with KCl at the time corresponding to fraction 4 and 12. The two peaks were referred to as S1 and S2 and occured approximately 2 fractions after the application of KCl due to the buffer volume contained in the tubes feeding into the incubation chamber. Values of the radioactivity eluted are expressed as fractional release which is the ratio of the radioactivity released in a particular fraction divided by the total amount of radioactivity contained in the tissue immediately prior to that fraction. The variation in the value of S1 of the two profiles shown in Fig 1, both induced by KCl alone, reflects the variation in amount of tissue present in each of the elution chambers and illustrates the need for utilising the ratio of the two peaks (S2/S1) of each elution profile as a mean to detect the effect of the modulating drug.

### Morphine modulation of KCl induced [^3^H]GABArelease

**Figure 2:**
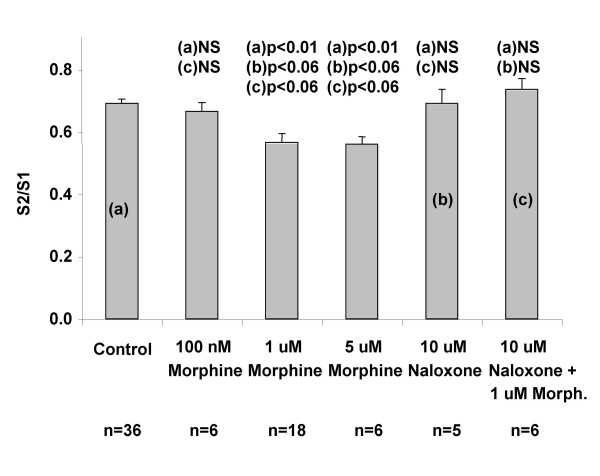
Effect of morphine on KCl-stimulted [^3^H]GABA release. The ratio of the integration of the peaks S2 over S1 was calculated for each elution profile. Morphine (0.1, 1 and 5 μM) and naloxone (10 μM) alone or together were perfused as described in figure 1 but naloxone perfusion was started one fraction earlier. Data represent averages ± SEM. Labels on the x-axis indicate concentration of drugs present during S2. Statistical difference was calculated using one-way ANOVA. The data indicates that 1 and 5 μM morphine caused a statistically significant 16% reduction of [^3^H]GABA release as compared to control (a) (p < 0.01). This effect of morphine was blocked by the antagonist naloxone (c) which had no effect on its own (b).

The effect of different concentrations of morphine on KCl-induced [^3^H]GABA release is shown in Figure 2. Both 1 μM and 5 μM but not 100 nM morphine caused a significant decrease of [^3^H]GABA release from the inferior colliculus slices. The effect of 1 μM morphine was antagonised by the antagonist naloxone (10 μM) which was perfused from one fraction before the addition of morphine. The perfusion of naloxone alone did not cause a significant effect on [^3^H]GABA release. These data strongly indicate that morphine modulates the release of [^3^H]GABA via activation of opiate receptors. The reduction in [^3^H]GABA release calculated as the change in S2/S1 ratios in the presence and absence of morphine during S2 was 16% (p < 0.01). These data agree with previous reports on the presence of both GABA neurons and opiate receptors and peptides in the inferior colliculus [7]. In addition a functional inter-relationship is established between the two systems which could be of physiological significance.

### Specific role of μ opiate receptors

**Figure 3:**
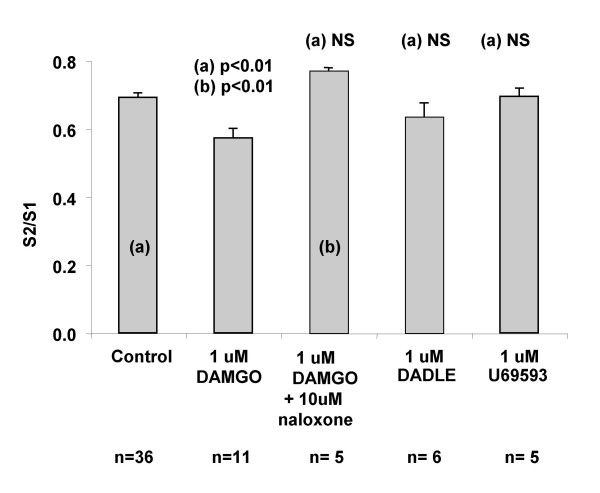
Effect of subtype-specific opoid peptides on KCl-stimulted [^3^H]GABA release. DAMGO, DADLE and U69593 (1 μM) were perfused as described for morphine in Figure 1. Data represent averages ± SEM. Labels on the x-axis indicate concentration of drugs present during S2. Only the μ-opioid receptor specific neuropeptide DAMGO produced a statistically significant effect (p < 0.01, One way ANOVA) on KCl-stimulted [^3^H]GABA release as compared to the controls (a) where no opiate drug was used. The effect of DAMGO was antagonised by 10 μM naloxone which was perfused from one fraction before the perfusion of DAMGO (b).

In order to establish which of the opiate receptor subtypes are involved in the modulation of the [^3^H]GABA release, morphine was substituted by either 1 μM DAMGO, DADLE or U69593 which specifically activate μ, δ and κ opiate receptors respectively (Fig. 3). Only DAMGO (1 μM) had a significant effect on [^3^H]GABA release, an effect that was again antagonised by naloxone. DAMGO, as well as morphine, reduced the amount of [^3^H]GABA release by 16% (p < 0.01) indicating that only μ opioid receptors participate in the regulation of GABA release. Higher concentrations of DAMGO (5 μM) did not have greater effects on [^3^H]GABA release (not shown). Data from our lab (unpublished) and from others [9,10] indicate that mRNA transcripts or receptor binding for all three opiate receptor subtypes are present in the inferior colliculus. Further work is required to establish the roles of the δ - and κ-opioid receptors in the inferior colliculus.

### Receptor desensitisation

**Figure 4:**
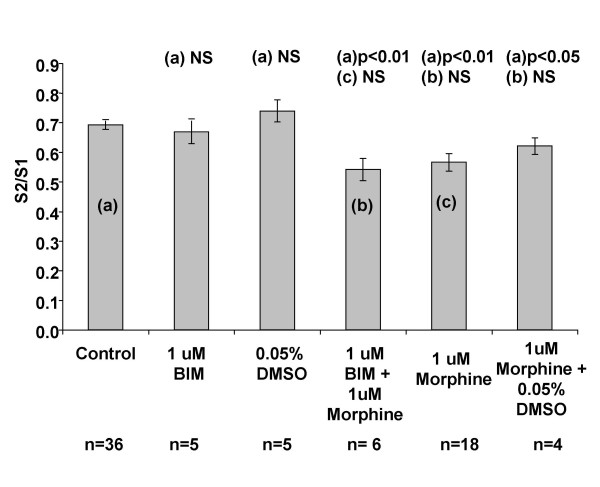
Effect of protein kinase C inhibitor bisindolylmaleimide I (BIM) on morphine modulation of [^3^H]GABA release. BIM is reported to reduce receptor desensitization and was used here to test whether morphine would elicit a bigger effect on [^3^H]GABA release in the presence of BIM. BIM is initially dissolved in DMSO whose effect on [^3^H]GABA release was also tested. BIM (1 μM) was present throughout the perfusion where indicated. Data represent averages ± SEM. Labels on the x-axis indicate concentration of morphine present during S2. The data show that morphine caused a reduction of [^3^H]GABA release as shown in Figure 2 but there was no statistical difference between experiments with or without BIM (One way ANOVA).

A possible explanation for the relatively low effect of opiate agonist on [^3^H]GABA release (16%) could be that during the exposure to opiate agonists, down-regulation of the opiate receptors may occur [19]. To address this possibility experiments were carried out in the presence of the protein kinase C inhibitor bisindolylmaleimide I (BIM). BIM has been shown to inhibit receptor desensitisation [20,21] and to reverse tolerance to opiate drugs which involves opiate receptor desensitisation. [22,23]. Because BIM is solubilised in DMSO additional control assays were carried out to check for the effect of the solvent. The results indicate (Fig. 4) that BIM had no effect on the extent of morphine inhibition of [^3^H]GABA release. While there is no direct proof that BIM had its reported effect on the tissue, the data indicate that receptor desensitisation may not be the cause of the relatively low percentage effect of morphine.

### Co-localisation of μ-opiate receptors and GABAergic neurons

**Figure 5:**
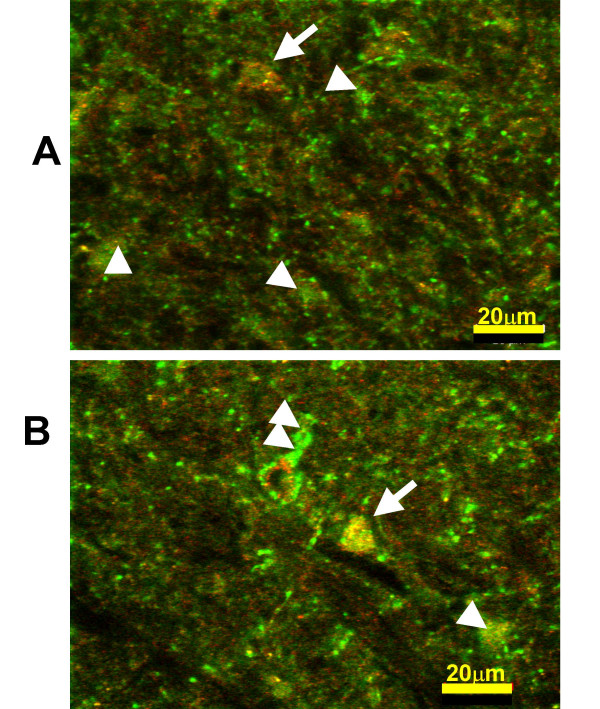
Confocal microscopy of cryosection of inferior colliculus slices stained with anti glutamic acid decarboxylase (green) and anti μ-opioid receptor antibodies (red). A : Central nucleus of inferior colliculus. B: Pericentral nucleus of inferior colliculus. Arrow head: example of GABAergic neurons not containing μ-opioid receptor (Green). Arrows: example of GABAergic neuron containing μ-opioid receptor (Yellow).

Another possible explanation for the small (16%) reduction of [^3^H]GABA release by opiate agonists may be the limited number of GABAergic neurons that express opiate receptors. To address this question inferior colliculi slices were double labelled with guinea pig antibodies against μ-opioid receptors and with rabbit antibodies against glutamic acid decarboxylase (the enzyme uniquely responsible for the synthesis of GABA) Species specific secondary antibodies conjugated to red and green fluorochromes allowed the detection of both antigens on the same slide (Fig. 5). Although these results were qualitative it was evident that staining of glutamic acid decarboxylase was more extensive than that of μ-opiate receptors and that only a few GABAergic neurons showed co-localisation of μ-opiate receptors. These data are consistent with the proposal that only a sub-population of GABAergic neurons are under the influence of opiate receptors. Establishing the nature of the GABAergic neurons that express opiate receptors will be an important task in understanding the role of opiate signalling in the inferior colliculus.

## Conclusions

This study has demonstrated that in the rat inferior colliculus slices opiate agonists can inhibit KCl-induced [^3^H]GABA release via activation of the μ-opiate receptor subtype. The amount of [^3^H]GABA released in presence of opiate agonists was 16% lower than in control slices. This relatively low level of decrease is probably not due to receptor desensitization occurring during the assay but rather to a relatively small population of GABAergic neurons in the inferior colliculus expressing μ-opiate receptors. The small effect of the opiate compounds could also indicate that modulation of GABA release is not their major role, but it could still be of physiological significance.

Together with its reported role in audiogenic seizures and aversive behaviour, the inferior colliculus is an important neuronal centre for auditory processing containing both ascending and descending fibres. The identification of the role of opiate peptides and possibly other modulatory system in the inferior colliculus and other areas of the auditory pathway may allow a better understanding of the mechanism of the hearing system and possibly offer a target for therapeutic intervention in hearing dysfunction. Alternatively, elucidation of the role opiate peptides in the inferior colliculus could provide information about regulation of audiogenic seizures and aversive behaviour.

## Methods

### Materials

Opiate agonist and antagonists, (Morphine, DAMGO [d-Ala(2), N-Me-Phe(4), Gly(5)-ol]-enkephalin, DADLE [D-Ala2, D-Leu5]-enkephalin, U69593 and naloxone were purchased from SIGMA, UK. Antibodies against μ-opiate receptor AB1774 (guinea pig polyclonal) and glutamic acid decarboxylase AB1511 (rabbit polyclonal) and species specific pre-absorbed secondary antibodies (donkey anti rabbit IgG FITC and donkey anti guinea pig IgG rhodamine) were purchased from Chemicon UK. Both antibodies were raised against synthetic peptides, and have been used in several immunocytochemical investigations of rat tissue. [24,25] AB1511 recognises the two isoforms of the enzyme in a Western blot (65/68 KDa) while antibody AB1511 recognises μ-opiate receptors immunocytochemically in the same tissues as other simlilar antibodies and by insitu hybridisation (Chemicaon data sheets, ) Krebs carbonate buffer: NaCl 118 mM, KCl 4.84 mM, CaCl_2 _2.4 mM, NaHCO_3 _25 mM, MgSO_4 _1.8 mM KH_2_PO_4 _1.2 glucose 9.5 mM.

### Animals

Sprague Dawley rats, approximately 200 g, were obtained from UCL Biological Services. All animal experiments were carried out in accordance to the Animal (Scientific Procedure) Act 1986, UK.

### Slices preparations

Rats were stunned and killed by cervical dislocation. The skull was opened and the whole brain removed. The inferior colliculus was dissected out by two coronal transections, the first between the cerebellum and the inferior colliculus and the second between the inferior colliculus and the superior coliculus. The slice was placed horizontally and medullar tissue ventral to the inferior colliculus was removed. The inferior colliculus was then placed on a tissue chopper and sliced into 250 μm coronal sections. Individual slices were separated under a dissecting microscope in Krebs buffer.

### Neurotransmitter release

As previously described, slices were incubated in 5 ml oxygenated (95% 0_2 _/ 5% CO_2_) Krebs buffer containing GABA transaminase inhibitor aminooxyacetic acid (100 μM) at 32°C for 5 min [26]. [^3^H]GABA was added to give a final concentration of 11 nM and incubated in a shaking water bath for 30 min. Slices were distributed into 6 superfusion chambers between filter papers (Brandel Superfusion System) and perfused at 0.5 ml/min with oxygenated Krebs buffer. Following a 30 min perfusion required to reach a steady state (non-stimulated) [^3^H]GABA release, 2 min fractions (1 ml) were collected. In order to evoke sub-maximal GABA release the slices were perfused for 2 min with 25 mMKCl at 6–8 min and 22–24 min of the fractionation time (fraction 4 and 12). At the end of each experiment (17 fractions) the tissue and the filter papers were collected and incubated with 500 μl Soluene-350 for 20 min and neutralised with 200 μl of glacial acetic acid. Scintillant (Packard, Ultima Gold, 3 ml) was added to all tissue samples and eluted fractions and the radioactivity was measured by scintillation counting (Wallac 1409). Each drug treament was repeated in several experiments as indicated in the figures and the ratios of the S2/S1 peaks were averaged. Statistical significance of the effect of treatments was analysed by one way ANOVA using Excel (Microsoft, USA)

### Immunocytochemistry

Inferior colliculi were dissected as decribed above and fixed in 4% paraformaldehyde in phosphate buffer saline (PBS: 137 mM NaCl, 2.7 mM KCl, 4.3 mM Na_2_HPO_4_.7H_2_O, 1.4 mM KH_2_PO_4 _pH 7.3) for 1 hour, washed 3 times in PBS and incubated overnight at 4°C in 30%sucrose in PBS. Coronal sections (20 μm) were cut with a cryostat and collected on poly-lysine coated glass slides and allowed to dry. The sections were blocked in 10% normal donkey serum diluted in PBS containing 0.25% bovine serum albumin and 0.1% Triton X-100 (PBS-A) for 30 min at room temperature. Subsequently, they were incubated for 12 hours at 4°C with the combination of primary antibodies (rabbit anti GAD and guinea pig anti mu opioid receptor) diluted 1:500 in PBS containing 1% bovine serum albumin and 0.3% Triton X-100 (PBS-B).. Slides were washed 3 × 10 min with PBS-B and incubated with secondary antibodies diluted 1:200 in PBS-B, for 2 hours at room temperature. The secondary antibodies used were donkey anti rabbit conjugated with fluorescein (AP182F) and donkey anti guinea pig conjugated with rhodamine (AP182R). Finally, the sections were rinsed in PBS-B for 10 min and in PBS for 2 × 10 min and then mounted in Citifluor (Agar). The immunoreactivity was visualized under the confocal microscopy (LSM 510 META Carl Zeiss, Germany).

## Authors' contributions

WT carried out the majority of the experiments, NJ carried out initial experiments and established assay conditions, JC provided expertise in neurotransmitter release studies and participated in the design of the study and analysis of the data, PP provided expertise in the double labelling studies, HD, AF and PG participated in the design of the study and analysis of the data, SOC conceived of the study and participated in its design and coordination. All authors read and approved the final manuscript.
